# Implementation of Sleep-Enhancing Strategies During Tapering in Elite Swimmers

**DOI:** 10.3390/sports14090412

**Published:** 2026-09-18

**Authors:** Quentin Bretonneau, Antonio Morales-Artacho, Robin Pla, Laurent Bosquet

**Affiliations:** 1 Laboratoire MOVE (UR 20296), Université de Poitiers, Faculté des Sciences du Sport, 86073 Poitiers, France; 2 Laboratoire Sport, Expertise and Performance (EA 7370), Institut Français du Sport (INSEP), 75012 Paris, France; 3 Fédération Française de Natation (FFN), 92110 Clichy, France

**Keywords:** pre-taper fatigue, sport performance, training load, sleep

## Abstract

This study examined whether performance changes following a taper incorporating sleep-enhancing strategies differed according to the pre-taper fatigue level in elite swimmers. Physiological, psychological, and biomechanical parameters were assessed in twenty-seven elite swimmers at two time points to estimate pre-taper fatigue: T0 and T1 (10 and 3 weeks before the main competition, respectively). Race performance was officially recorded at T1 and during the main competition. Sleep-enhancing strategies included two sleep education sessions, five consecutive evenings of cryostimulation, and the use of a mattress topper with high thermal conductivity. Statistical significance was set at *p* ≤ 0.05. Thirteen swimmers (17 ± 2 y) were classified as acutely fatigued (AF) and fourteen (17 ± 1 y) as functionally overreached (F-OR). From T1 to competition, performance changes were not different between groups (AF: +1.2 ± 1.6% vs. F-OR: +0.4 ± 2.3%). A total of 82% of swimmers in the AF group and 67% in the F-OR group achieved a performance improvement greater than 0.5% (*p* > 0.05). Following a tapering period incorporating sleep-enhancing interventions, no significant differences were observed between AF and F-OR swimmers in the magnitude of performance change or in the proportion of swimmers exhibiting a performance improvement greater than 0.5%.

## 1. Introduction

According to the mathematical model proposed by Banister and Fitz-Clarke [1], performance in sports such as swimming is largely determined by the difference between physical fitness and fatigue levels. From this perspective, the objective when preparing for an important competition, such as the Olympic Games, is to achieve the most favorable possible fitness–fatigue balance. This means reducing accumulated fatigue to the greatest extent possible while ensuring that the physical adaptations developed through training are retained. To achieve this, coaches generally incorporate a taper into the final phase of preparation, during which the overall training load is deliberately lowered for approximately 2–4 weeks before competition. Bosquet et al. [2], in a meta-analysis of tapering strategies, identified a progressive reduction in training volume of 40–60% across two weeks, combined with an unchanged exercise intensity and frequency, as the approach producing the most favorable outcomes in high-performance athletes. On average, this type of taper was associated with a 1.9% increase in performance. Although relatively small in absolute terms, such an improvement may be decisive at the highest competitive level. Indeed, the smallest performance change likely to meaningfully increase the chances of winning a medal has been estimated to correspond to roughly one-third of the normal variation observed in competition, with this threshold being approximately 0.5–1% for swimming [3,4]. Importantly, however, the magnitude and direction of the performance response to tapering are not identical among athletes, as demonstrated by the inter-individual variability reported by Bosquet et al. [2]. The fatigue accumulated before the taper begins has been proposed as a potential determinant of these differences in response. Initial support for this proposition came from mathematical simulations [5], while subsequent experimental evidence has been obtained in endurance disciplines [6,7], team sports [8], and, more recently, in elite swimmers [9].

In this previous study, we observed an average performance gain of 1.8% in swimmers who began the taper with acute fatigue level, whereas those who started the taper with elevated fatigue exhibited a performance decrement of about 0.5%. These findings suggest that a traditional taper (one based solely on reducing training load) may be insufficient to achieve full recovery and, consequently, may not allow for performance improvement following the taper. In alignment with this, these findings underscored the potential interest of optimizing sleep during the taper phase. Indeed, sleep is recognized as a cornerstone of recovery: beyond its general restorative effects, it promotes anti-inflammatory, antioxidant, and anabolic processes that facilitate cellular and tissue repair, thereby enhancing recovery [10].

Therefore, the aim of this study was to compare the performance changes (the primary outcome) following a taper incorporating sleep-enhancing strategies in swimmers with high versus acute pre-taper fatigue, the latter group, which could be considered a reference group within the context of the current study. We hypothesized that incorporating sleep-enhancing strategies into the taper would allow swimmers with higher pre-taper fatigue to achieve performance changes that were not significantly different from those observed in swimmers with lower initial fatigue.

## 2. Materials and Methods

### 2.1. Protocol

**Figure 1:**
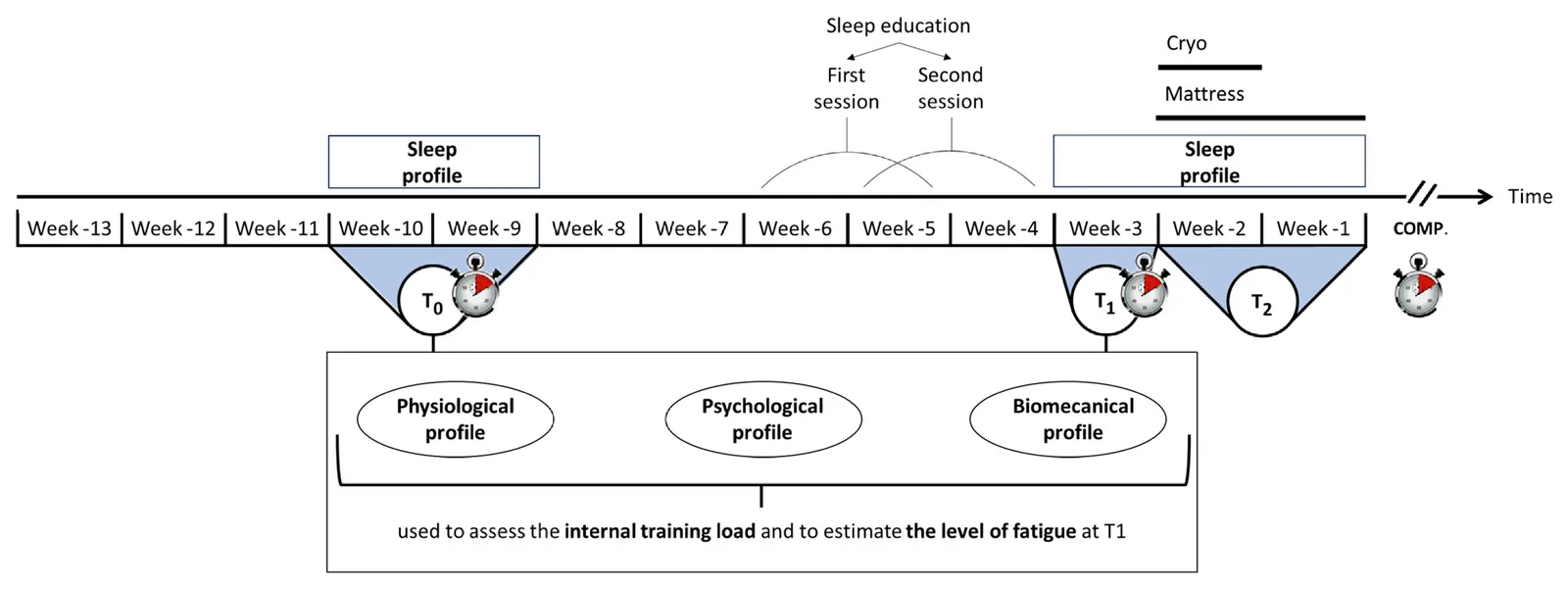
Experimental protocol. T0: the baseline period; T1: the week preceding the taper period; T2: the taper period.

This study extends our previous work investigating the impact of pre-taper fatigue on taper responses in elite swimmers. Whereas the previous study identified poorer sleep characteristics and impaired taper responses in functionally overreached swimmers [9], the present investigation evaluates whether a taper incorporating sleep-enhancing interventions may attenuate these differences. Thus, as in our previous study [9], the current study was conducted across three national training centres of the French Swimming Federation. For each centre, the protocol started 13 weeks before the competition of interest and ended on the evening of the final day of that competition (Figure 1).

Physiological, psychological and biomechanical profiles were evaluated twice to estimate the pre-taper level of fatigue: at T0 (a two-week baseline period scheduled ten and nine weeks before the competition of interest) and T1 (a one-week period scheduled three weeks before the competition). Sleep profile was evaluated during these two periods and during the two weeks preceding the competition (T2). Performance was evaluated at T0 and T1 (±7 days), and during the competition of interest. Based on changes in physiological, psychological, and biomechanical profiles between T0 and T1, participants were retrospectively classified into two groups with distinct levels of fatigue prior to tapering: acute fatigue (AF) and functional overreaching (F-OR). Interventions known to improve sleep quantity and quality were scheduled between T0 and T1 and during T2.

The Ile-de-France Committee for the Protection of Persons (i.e., the French Ethics Committee for Biomedical Research) reviewed and approved the study protocol (N° ID-RCB: 2020-A03559-30 and N° CNIL/MR-001: 2220344 [9]). Moreover, the protocol was conducted according to recognized ethical standards and national/international laws. All participants (or the legal representatives for the minor participants) gave their written informed consent to participate in the study.

### 2.2. Participants

Assuming the use of an independent samples *t*-test (swimmers in acute fatigue vs. in functional overreaching), the a priori power analysis indicated that eight swimmers per group would be needed to have an 80% chance of obtaining a significant difference (*p* < 0.05) in taper-induced gain in performance (decrease in race time). According to this analysis based on the results of Aubry et al. [6], at least 16 swimmers had to be recruited. To account for group distribution uncertainty, potential withdrawals, or missing performance data, 30 participants were recruited [9].

Thirteen male and seventeen female swimmers were included in the study. All of them were considered Tier 3 swimmers according to the classification by McKay et al. [11] (highly trained/national). One male and two female swimmers were excluded due to injury and inability to follow the protocol fully. The final sample size was twelve male swimmers (age: 17.6 ± 1.4 years old; body height: 1.81 ± 0.06 m; body mass: 69.5 ± 7.1 kg; personal best performance: 89.9 ± 2.3% of the world record) and fifteen female swimmers (age: 16.8 ± 1.6 years old; body height: 1.70 ± 0.04 m; body mass: 59.8 ± 8.4 kg; personal best performance: 87.3 ± 3.2% of the world record). One athlete was excluded from the analyses involving T2 data due to an 80% increase in training load observed during the taper period.

### 2.3. Procedures

#### 2.3.1. Performance

Performance was assessed using race times recorded during official competitions scheduled and certified by the French Swimming Federation. Race times were obtained using an Omega timing system (SwissTiming Ltd., Corgémont, Switzerland) and subsequently retrieved from the Federation’s official website. For each swimmer, the analysis considered the stroke and distance combination for which the best performance achieved during the study protocol was the closest to the corresponding world-record time.

#### 2.3.2. External Training Load

External training load was monitored throughout the 10-week period preceding the competition. Following every swimming or dryland training session, swimmers entered the session duration (min) and their rating of perceived exertion (RPE), assessed using the CR-10 scale, in their individual logbook. Training load was subsequently calculated as the product of session duration and RPE [12] and reported in arbitrary units. For swimming sessions, three variables were considered: training load (SS_load_), session duration (SS_duration_), and RPE (SS_RPE_). The corresponding variables were also determined for dryland sessions (DS_load_, DS_duration_, and DS_RPE_). In addition, combined-session variables (CS_load_, CS_duration_, and CS_RPE_), calculated by summing the swimming and dryland components, were included in the analyses. During the entire experimental period, coaches were asked to preserve their usual approach to regulating external training load, including the number, duration, and intensity of swimming and dryland sessions during both training and tapering periods. From T0 to T1, the weekly training load (i.e., the average training load from Monday to Sunday) was analysed.

The changes in training load, session duration, and RPE during the taper were additionally examined. These changes were expressed as follows:

$$\%\;change=\frac{A-\left(B*14\right)}{B*14}*100$$

where A corresponded to the sum of the values recorded on each day of the 14-day taper, whereas B represented the mean daily value calculated over the three weeks preceding the taper [9].

#### 2.3.3. Internal Training Load

At T0 and T1 (Figure 1), physiological, psychological, and biomechanical profiles were evaluated to characterize the swimmers’ internal responses to training. The procedures were identical to those described previously by Bretonneau et al. [9] and are only briefly summarized here.

(a) Physiological profile.

The physiological profile was established using heart rate (HR) measurements obtained at rest, during a standardized constant-intensity submaximal swimming test, and during post-exercise recovery. Heart rate was continuously recorded using a wrist-worn monitor based on photoplethysmography (Polar Unite, Polar Electro Oy, Kempele, Finland). The device was placed on the non-dominant wrist and tightened sufficiently to ensure appropriate recording during exercise [13].

Resting HR was recorded every morning throughout T0 and T1. Each measurement lasted 5 min and was performed in a dark environment before the swimmer left bed. Participants remained in a lying position and were instructed to keep movements to a minimum both before and throughout the recording period.

Exercise HR was assessed daily during T0 and T1. For each assessment, swimmers completed a 6 min swim while attempting to cover an identical distance. The target distance was determined during the first three days of T0. This distance corresponded to an RPE of 3 on the CR-10 scale and an HR ranging from 120 to 160 beats·min^−1^. Once determined, the target distance remained unchanged and was prescribed for every subsequent assessment throughout the protocol. Heart rate recordings were included in the analysis only when the distance completed was within ±10 m of the predetermined target.

Immediately after the 6 min swim, participants remained upright, leaning against a swimming lane, for 3 min and were instructed to minimize their movements. Post-exercise HR recovery was quantified as the difference between HR recorded immediately at the end of exercise and HR recorded 60 s later (∆60).

These measures were selected for analysis based on their previously demonstrated reproducibility [14,15,16] and their sensitivity to overreaching [17,18].

(b) Psychological profile.

The psychological profile was characterized using the Profile of Mood States questionnaire (POMS; [19]). In addition, resilience was evaluated using the Connor-Davidson Resilience Scale (CD-RISC; [20]).

The POMS comprises 65 items and assesses six distinct mood dimensions: vigor, depression, fatigue, anger, anxiety, and confusion. An energy index (EI) was calculated as the difference between vigor and fatigue scores. Because vigor, fatigue, and EI have been shown to be sensitive to performance changes induced by overload and tapering, these three variables were retained for subsequent analyses [18]. The POMS was completed once at T0 and once at T1.

Resilience was assessed using the 25-item CD-RISC, which has been validated in elite athletes [21]. This questionnaire was completed once, at the beginning of the protocol, and was used to assess the degree of confidence that could be placed in the results obtained from the POMS questionnaire [22].

(c) Biomechanical profile.

The biomechanical profile was obtained from force measurements collected during a 10 s tethered front-crawl swimming test. At T0, the test was completed twice per day on three consecutive days, whereas at T1 it was performed twice on a single day. Each testing session began with a standardized 20 min warm-up, followed by two 10 s maximal-intensity front-crawl tethered swimming trials performed without breathing. A 5 min period of passive recovery separated the two trials.

The recorded signal represented the force generated by the swimmer over time. Individual swimming cycles were subsequently identified manually, with one cycle corresponding to the force generated by one action of each arm. In accordance with previous recommendations, the first cycle was excluded from the analysis [23]. Mean force and mean impulse, defined as the time integral of force, were then calculated from the remaining cycles and averaged across the two trials performed on each testing day.

#### 2.3.4. Sleep Profile

Chronotype was assessed once, at the beginning of the protocol, using the 19-item Horne and Ostberg Morningness–Eveningness Questionnaire (16–86 score range; [24]).

Sleep habits were assessed once at T0 using the Pittsburgh Sleep Quality Index (PSQI, 0–21 score range; [25]).

Subjective sleep quality was assessed each morning during T1 and T2 with the six-item Spiegel questionnaire (0–30 score range; [26]). A few minutes after awakening, swimmers completed the questionnaire directly within their logbook. They also reported their bedtime, defined as the time at which they went to bed irrespective of whether they immediately fell asleep, their lights-out time, corresponding to the time at which they switched off the lights or smartphone with the intention of sleeping, and their wake-up time.

Nocturnal movements were additionally quantified using an accelerometer worn every night during T1 and T2 (ActiGraph wGT3X-BT, ActiGraph LLC, Pensacola, FL, USA). The device was worn on the non-dominant wrist. Movement index and fragmentation index were also derived from these recordings.

Core body temperature was assessed during T1 and T2 for at least one night in the middle of the week. Under the supervision of an experimenter, swimmers ingested an encapsulated temperature sensor at approximately 4:00 p.m. (e-Celsius Performance, BodyCap, Caen, France).

### 2.4. Categorization of Fatigue

The swimmers were classified as either acutely fatigued (AF group) or functionally overreached (F-OR group) according to the changes observed between T0 and T1 across the different profiles and their underlying variables. The classification procedure followed the strategy previously established by our research team [8,9,27], which takes into account both the number and magnitude of negative changes. Specifically, swimmers were assigned to the AF group when several small-to-moderate negative changes were observed, and no more than one large negative change was present; a negative EI was considered a negative change within this classification. In contrast, swimmers were categorized as F-OR when at least one large negative change was identified in two or more distinct profiles.

For variables assessed repeatedly at both T0 and T1 (e.g., heart rate), the magnitude of the change was quantified using Cohen’s d. For variables measured repeatedly at T0 but only once at T1 (e.g., force produced during the tethered swimming test), the magnitude of change was determined using the Z score. For variables assessed once at T0 and once at T1 (e.g., vigor), the magnitude of change was evaluated using the smallest worthwhile change (SWC). The mathematical procedures used to calculate Cohen’s *d*, Z score, and SWC have been reported previously [9,27].

### 2.5. Sleep-Enhancing Strategies

#### 2.5.1. Sleep Education

Two one-hour group interventions were scheduled between T0 and T1 for each centre. These in-person interventions were led by a consortium member with expertise in the field and were based on content that has been previously demonstrated to improve sleep outcomes [28,29]. The first intervention, scheduled two to three weeks prior to the taper period, focused on general aspects of sleep (mechanisms and benefits, especially for performance in high-level swimmers). The second intervention, scheduled one to two weeks prior to the taper period, addressed the detrimental effects of insufficient or poor-quality sleep. This session also covered topics such as daytime sleepiness and the role of napping. Additionally, a practical exercise was offered to facilitate falling asleep, which included techniques such as cardiac coherence, breathing focus, and relaxation.

#### 2.5.2. High Thermal Conductivity Mattress

During the fourteen nights preceding departure for the competition of interest, all swimmers benefited from a mattress topper with high thermal conductivity (Bultex, Noyen-sur-Sartre, France). The mattress topper was entirely composed of foam (density: 40 kg/m^3^) and incorporated a flush-mounted high thermal capacity gel sheet (density: 1000 kg/m^3^; dimensions: 80 × 120 × 2.5 cm) to dissipate body heat during sleep through conductive transfer. The mattress was encased in a cover with a 540 g/m^2^ density, resulting in thermal resistances of 1.18 m^2^·K·W^−1^ [30,31]. The bedding was not modified, and the swimmers continued to use their duvet and pillow.

#### 2.5.3. Cryostimulation or Contrast Baths

Immediately after T1, twenty swimmers were exposed to cryostimulation every day for five days. The sessions were scheduled between 6:00 pm and 8:00 pm, within an hour after the end of training. Two different systems were used. The first system, used by nine swimmers, was a whole-body cryostimulation (WBC) chamber [32]. The second system, used by eleven swimmers, was a cabin designed for partial-body cryostimulation (PBC, excluding the head). Each WBC session consisted of a 30 s exposure in a first chamber at −20/−25 °C (perceived temperature: −50 °C) and a 3 min exposure in a second chamber at −45/−50 °C (perceived temperature: −110 °C) (AuroreConcept, Noisiel, France). Each PBC session consisted of a 3 min exposure in the cabin cooled at −110 °C by liquid nitrogen (LaMobile, Cryo-Soft, Dijon, France). At each session, the swimmers were required to wear dry underwear and were equipped with gloves, slippers, a cap, and a surgical mask. The remaining seven swimmers performed a contrast bath protocol (partial body immersion excluding the head) consisting of seven cycles of two minutes in hot water (40 °C) followed by two minutes in cold water (8 °C).

### 2.6. Statistical Analysis

Descriptive statistics were obtained using standard procedures, with results expressed as means and standard deviations. The assumption of Gaussian distribution was evaluated using the Shapiro–Wilk test. Differences between the AF and F-OR groups were examined using an independent-samples *t*-test or, when the assumptions for parametric testing were not satisfied, a Wilcoxon test. These analyses addressed three null hypotheses: (1) no difference existed between the AF and F-OR groups at T0; (2) external training load accumulated during the taper did not differ between groups; and (3) the change in performance from T1 to the competition of interest did not differ between groups.

To examine differences across groups and periods, a two-way factorial analysis of variance (group x period), with repeated measures on the period factor, was conducted. For core body temperature and accelerometry-derived counts, a three-way analysis of variance (group x period x time) was additionally performed. The assumption of compound symmetry, or sphericity, was assessed using Mauchly’s test. Whenever sphericity was violated, the F-test significance level was corrected using the Greenhouse–Geisser procedure for epsilon values below 0.75 and the Huynh–Feldt procedure for epsilon values above 0.75, thereby limiting the risk of type I error. Pairwise comparisons were subsequently adjusted using the Bonferroni post hoc procedure.

Effect sizes were expressed as Hedges’ g (g) or Cohen’s d (d) and interpreted as small (0.2 < g or d < 0.5), moderate (0.5 ≤ g or d < 0.8), or large (g or d ≥ 0.8). Associations between variables and/or their changes were examined using Pearson’s product-moment correlation or, when appropriate, Spearman’s rank-order correlation. Correlation coefficients greater than 0.90 were interpreted as very high, those ranging from 0.70 to 0.89 as high, and values between 0.50 and 0.69 as moderate [33]. The proportion of swimmers in the AF and F-OR groups who exhibited performance improvements exceeding 0.5% was compared using Fisher’s exact test. The significance level was set at *p* < 0.05. To control for multiple comparisons, a Bonferroni correction was applied within each family of outcomes. Accordingly, the significance threshold was adjusted to *p* < 0.01 for the sleep diary outcomes and to *p* < 0.007 for the accelerometer-derived parameters. All the calculations were made with Statistica (StatSoft, Tulsa, OK, USA).

## 3. Results

### 3.1. AF and F-OR Groups

Individual variations in physiological, psychological, and biomechanical parameters between T0 and T1 are presented in Table 1. The characteristics of the AF and F-OR groups are detailed in Table 2.

### 3.2. Performance

**Figure 2:**
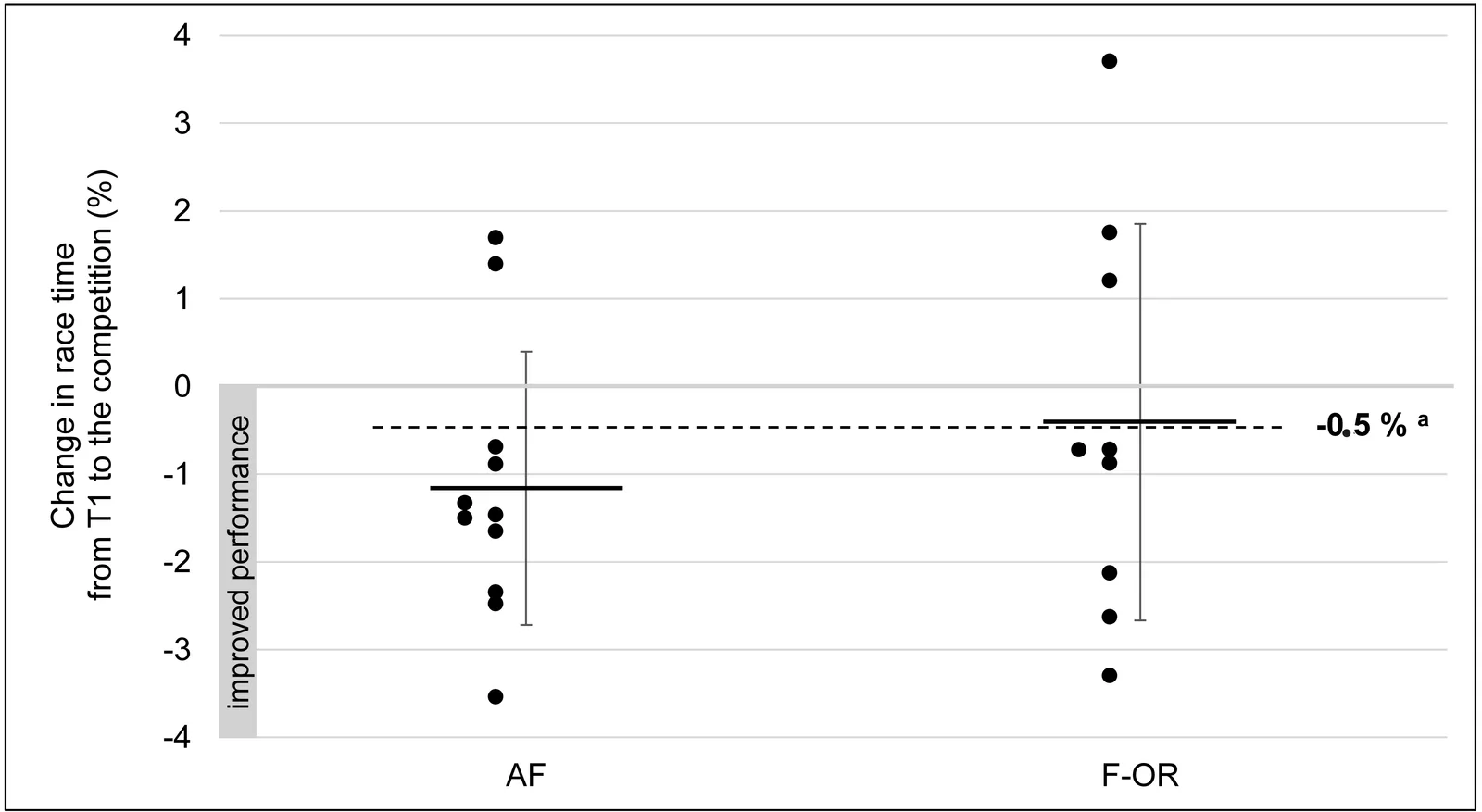
Mean and individual changes in race time from T1 to the competition in acute fatigue (AF) and functional overreaching (F-OR) groups. ^a^, the minimal performance improvement that can substantially affect the probability of winning a medal.

Descriptive analysis showed that performance improved by 1.2 ± 1.6% in the AF group and by 0.4 ± 2.3% in the F-OR group from T1 to competition. Statistical analysis revealed no significant difference in performance change between the AF and F-OR groups (n AF = 11 swimmers; n F-OR = 9 swimmers; *p* = 0.39; d = 0.4; difference: −0.8 percentage points; 95% CI: −2.7 to 1.2). A total of 82% of swimmers in the AF group and 67% in the F-OR group achieved a performance improvement greater than 0.5% (*p* = 0.62, Figure 2).

### 3.3. Training Load and Taper Characteristics

The overall training load did not differ between groups at T0 and remained relatively stable over this two-week period (597 ± 255 a.u. in the first week and 589 ± 187 a.u. in the second week). From T0 to T1, the coaches structured the training program into a five-week mesocycle including three microcycles. The first and third microcycles consisted of two weeks of progressive loading, while the second microcycle consisted of a one-week deloading phase. No difference in training load was observed between groups during these microcycles. In the first one, the weekly training load peaked at 802 ± 374 a.u. before returning to 587 ± 187 a.u. in the second microcycle. In the third microcycle, the weekly training load peaked at 863 ± 366 a.u. during the week preceding T1 and was significantly higher than at T0.

**Figure 3:**
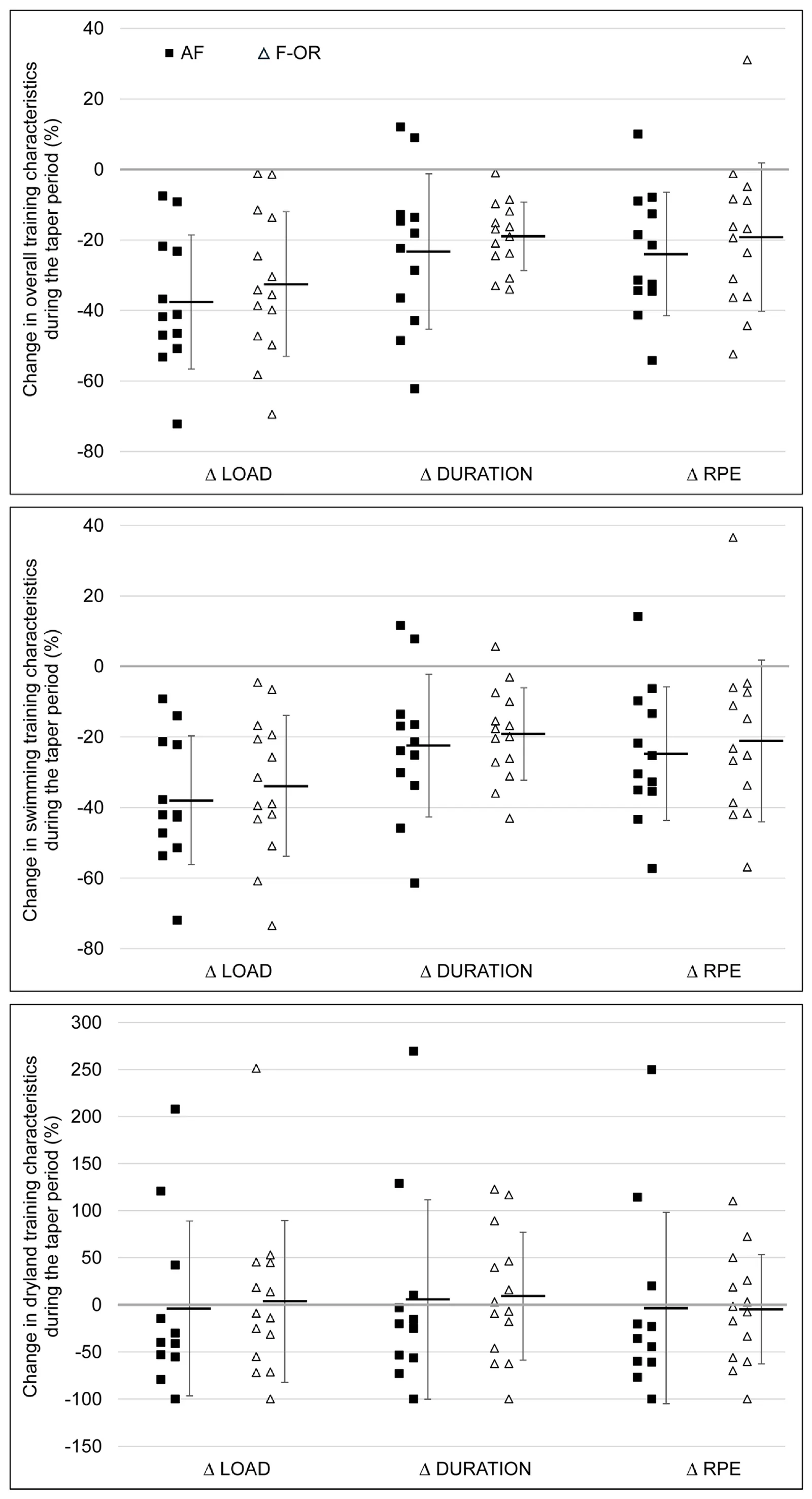
Mean and individual changes in training characteristics (load, duration and RPE) during the taper period. **Top**: overall training; **Middle**: swimming training; **Bottom**: dryland training. RPE: rate of perceived exertion.

The changes in overall training load over the two-week tapering period did not differ between AF and F-OR groups (Figure 3). Similar results were observed for overall training duration, overall training RPE, swimming-related training load and dryland-related training load.

### 3.4. Sleep Outcomes

As shown in Table 3, sleep latency decreased between T1 and T2 (*n* paired observations: 25; *p* < 0.001; g = 1.6; difference = 7.8; 95% CI 5.8 to 9.8). The kinetic analysis of movement counts did not reveal a significant difference between groups, nor between periods.

### 3.5. Nocturnal Core Body Temperature

**Figure 4:**
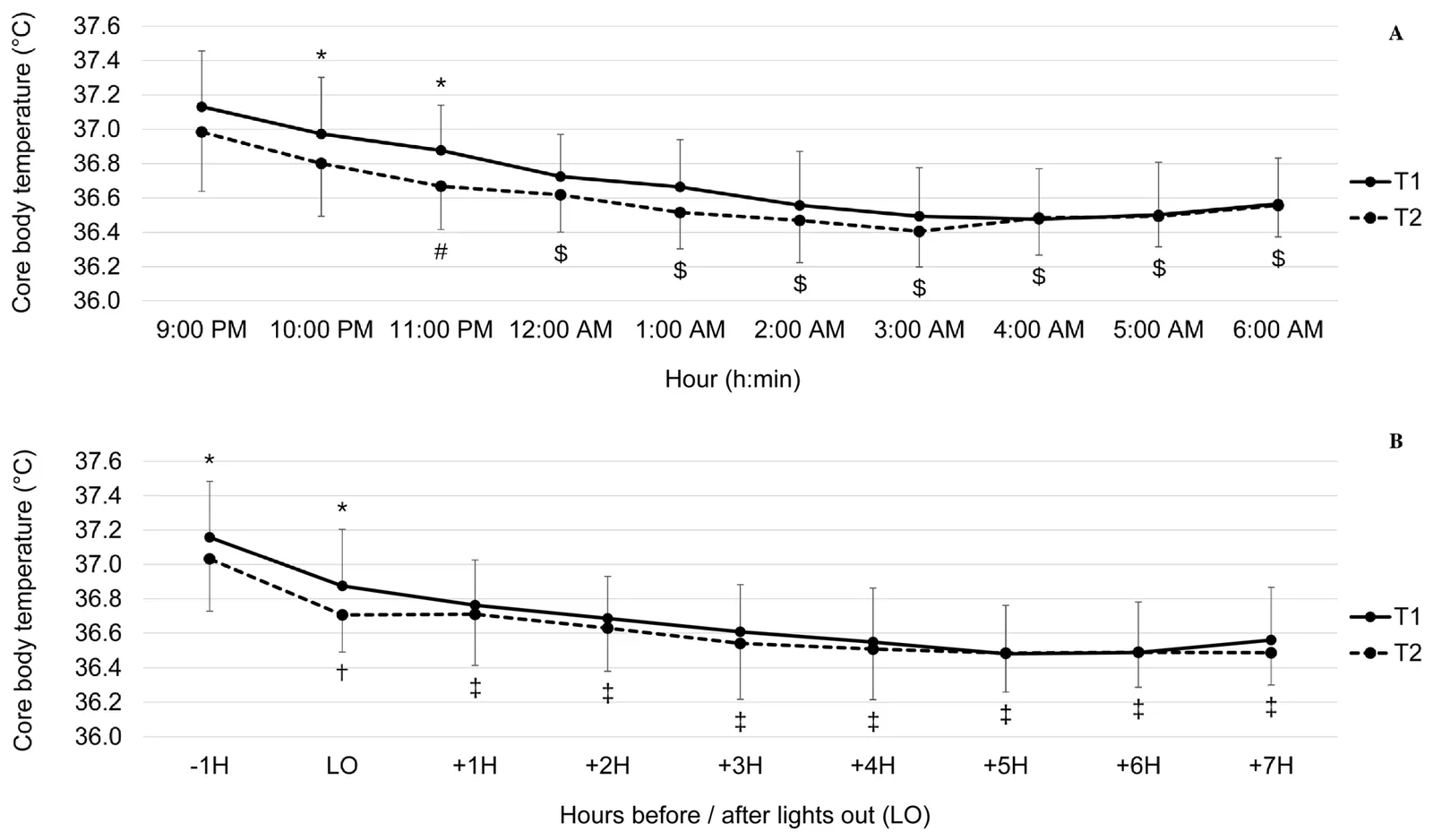
Core body temperature from 9:00 PM to 6:00 AM (**A**) and from 1 h before lights out (LO) to 7 h after (**B**) at T1 and T2. *, different from T2 for the corresponding time point; #, different from 9:00 PM only, regardless of the period; $, different from 9:00 PM and 10:00 PM, regardless of the period; †, different from −1H, regardless of the period; ‡, different from −1H and LO, regardless of the period. T1: the week preceding the taper period; T2: the taper period.

The kinetics of core body temperatures measured at T1 and T2 are detailed in Figure 4 (from 9:00 PM to 6:00 AM and from one hour before lights out to seven hours after). The temperatures at 10:00 PM and 11:00 PM were lower at T2 compared to T1. Furthermore, the temperatures at lights out and one hour prior were lower at T2 than at T1 (n paired observations: 22 at each time point). In both periods, temperature significantly decreased during the hour preceding lights out. Additionally, the nadir of the temperature kinetic was lower in the F-OR group compared to the AF group (36.18 ± 0.15 °C vs. 36.40 ± 0.21 °C, d = 1.1). The time required to reach this nadir from lights out was shorter at T2 than at T1 (−73 ± 137 min, g = 0.5).

## 4. Discussion

The aim of this study was to compare the performance changes following a taper incorporating sleep-enhancing strategies in swimmers with acute versus high pre-taper fatigue. The findings revealed no significant difference between groups.

### 4.1. Pre-Post Tapering Performance Changes: Comparison Between the AF and F-OR Groups

In the current study, both the AF and F-OR groups increased their performance on average following taper incorporating sleep-enhancing strategies (+1.2 ± 1.6% and +0.4 ± 2.3%, respectively), with no difference observed between groups. In a previous study involving a conventional taper (characterized solely by a reduction in training load), a significantly different tapering response was observed between groups, with performance increasing in the AF group (+1.8 ± 1.4%) but declining in the F-OR group (−0.5 ± 1.6%, [9]). These findings suggest that taper incorporating sleep-enhancing strategies does not provide greater benefits than conventional tapering in improving performance among swimmers experiencing acute pre-taper fatigue. In contrast, it appears particularly relevant for swimmers exhibiting functional overreaching. This supports the hypothesis that swimmers experiencing a high level of fatigue before tapering require greater and more rapid recovery. For these swimmers, a reduction in training load alone may be insufficient to eliminate the accumulated fatigue. Instead, integrating sleep-enhancing interventions alongside load reduction may constitute a more effective strategy to facilitate complete recovery and optimize subsequent performance.

It is also worth noting that the changes in performance from pre-taper to competition were likely underestimated in the F-OR group in the current study, as one swimmer exhibited a performance decline of 3.7%, the greatest deterioration observed across both studies, with the second highest being a 2.8% decline. When this atypical case is accounted for separately, the mean performance change in the F-OR group shifts to a 0.9% improvement. Furthermore, analysis of individual responses within this group revealed that two-thirds of the swimmers achieved a performance improvement exceeding 0.5%, a threshold considered to represent the minimal enhancement required to substantially increase the likelihood of winning a medal in competitive swimming [4]. In contrast, in our previous study [9], only 25% of the F-OR swimmers surpassed this threshold following conventional tapering. It should be noted that, although these two studies differ notably in terms of the tapering strategy itself (i.e., a conventional taper versus a taper incorporating sleep-enhancing strategies), they also differ with regard to the participants included and the management of training load, especially during the tapering period (see the following paragraph).

### 4.2. Training Load Management

In the present study, as in the previous one [9], the experimenters did not prescribe training load to coaches before or during the tapering period. Rather, current evidence-based recommendations were provided, and the final tapering strategy was determined by the coach. The training load during the 10-day baseline period did not differ significantly between the AF and F-OR groups and remained stable throughout this phase. This consistency indicates that baseline measurements were collected under steady training conditions. Following this period, both groups engaged in a five-week mesocycle, during which no significant between-group differences in training load variation were observed. A marked increase in training load was recorded between the baseline and pre-taper phases, confirming the implementation of a development phase between these periods. During the tapering period, the reduction in overall training load did not significantly differ between the AF and F-OR groups. In a previous study, tapering recommendations were not fully met, with training load reductions of 30% in the AF group and only 23% in the F-OR group [9], both below the 40–60% reduction range typically recommended in the literature [2]. The present study showed a reduction of 38% in the AF group, aligning more closely with these guidelines, while the F-OR group exhibited a decrease of 32%, which, although slightly improved, remained below the recommended range.

### 4.3. Choice of Sleep-Enhancing Strategies

In the preceding investigation [9], we reported that, before the tapering phase, the F-OR group exhibited a reduced total sleep time, a trend toward greater sleep fragmentation, and higher daytime sleepiness compared to the AF group. Concurrently, the decline in core body temperature observed during the hour preceding bedtime was less marked in the F-OR group than in the AF group. Moreover, in both groups, bedtime and lights out time were delayed just before the taper period compared to baseline, and this was accompanied by an increase in mean core body temperature during sleep in the F-OR group. Based on these previous observations, we decided in the current study to focus our sleep enhancement strategies on healthy sleep habits and behaviours, thermoregulatory processes and naps to induce a phase advance, improve sleep quality, and reduce daytime sleepiness during the taper phase. This intent was further supported by our observation that both lights-out time and nocturnal core body temperature were associated with performance outcomes [9].

In this context, we sought to implement a complex intervention including several strategies that have been scientifically demonstrated to be effective in addressing the issues highlighted [34]. We also intentionally selected strategies that were readily available and feasible within sports training environments, thereby ensuring that our field-based approach remains practical, ecologically valid, and sustainable in the long term [34]. More specifically, among the sleep enhancement strategies implemented in the present study, a sleep education intervention was conducted, grounded in previously validated protocols, resulting in improved sleep latency and efficiency in young rugby players [29], and earlier bedtime and reduced daytime sleepiness in swimmers [28]. In addition, during fourteen consecutive nights, swimmers used a high thermal conductivity mattress topper placed atop their usual mattress. This strategy was adopted based on evidence from previous studies demonstrating lower nighttime core body temperature, increased slow-wave sleep, and improved subjective sleep quality when using such mattresses compared to standard ones [35,36,37]. Furthermore, swimmers underwent either evening body-cryostimulation (n = 19) or contrast bath (n = 7) sessions during the first five days of the tapering period, depending on the equipment available within their training centre. Among the 19 swimmers who underwent whole-body cryostimulation, nine were classified as AF and ten as F-OR. Among the seven swimmers who underwent the contrast bath intervention, three were classified as AF and four as F-OR. The implementation of cryostimulation sessions was based on evidence indicating that this intervention can reduce core body temperature before bedtime and improve both slow-wave sleep duration and perceived sleep quality (2,3,15,23). Similarly, the contrast bath protocol has been shown to exert beneficial effects on sleep, particularly by facilitating sleep onset [38]. No adverse events related to the intervention were observed.

### 4.4. Practical Recommendations/Applications

This study provides several practical recommendations, with the foremost being the assessment of swimmers’ fatigue level before the tapering phase. Such assessment enables the identification of individuals exhibiting signs of functional overreaching, which in turn allows for: (1) individualized tapering, with a greater reduction in training load for overreached swimmers, and (2) the implementation of targeted sleep-enhancing interventions during the taper, to accelerate recovery and optimize subsequent performance.

Regarding the second point, our findings offer guidance on incorporating sleep education and modulating thermoregulatory processes to enhance sleep-related recovery mechanisms. Although this study integrated a combined intervention approach, practical field applications may benefit from a more individualized strategy. This could be operationalized through the development of an individualized decision-making framework that identifies the most appropriate sleep-related interventions based on the specific needs or issues encountered. However, constructing such a framework can be challenging in applied settings, as it requires regular assessment of the athlete’s sleep profile and systematic evaluation of the effectiveness of all available strategies, while also considering the athlete’s adherence to these interventions. Therefore, the construction of this decision tree should be initiated as early as possible, developed progressively, and must account for both intrinsic and extrinsic changes in the athlete throughout their career. In order to examine sleep more holistically, it would also be relevant to consider the nutritional and psychological domains. This would enable us to propose targeted solutions to address issues arising within these components, thereby complementing the decision-making framework and enhancing the individualisation of interventions [27].

### 4.5. Limitations and Perspectives

Our findings indicate that pre-taper fatigue status did not influence the response to tapering when the taper incorporated sleep-enhancing strategies. This contrasts with our previous study, in which pre-taper fatigue status influenced the response to a more conventional taper. Therefore, an important limitation, which also represents a key avenue for future research, is the absence of a direct comparison between a conventional taper and a taper incorporating sleep-enhancing strategies according to swimmers’ pre-taper fatigue status. Such a design would allow a more direct evaluation of whether integrating sleep-enhancing strategies can mitigate the influence of pre-taper fatigue status and would strengthen the practical recommendations derived from these findings.

Another limitation relates to the pragmatic nature of our field-based intervention. To maximize ecological validity and ensure that the protocol could be implemented within the resources available at each training centre, we adopted a multicomponent sleep-enhancing package that was slightly adapted according to local facilities (e.g., cryostimulation or contrast bathing). By contrast, a laboratory-based experimental design would have required exactly the same intervention package to be delivered to all participants, thereby providing a higher level of methodological standardization. Nevertheless, the distribution of the different recovery modalities was, by chance, globally well-balanced between the AF and F-OR groups, making it unlikely that these minor differences substantially influenced the between-group comparisons.

## 5. Conclusions

Despite differences in pre-taper fatigue status, swimmers exhibiting acute fatigue and functional overreaching showed no significant differences in either the magnitude of performance change or the proportion of swimmers achieving a performance improvement greater than 0.5% following a taper integrating sleep-enhancing interventions. These findings suggest that, under the conditions investigated, pre-taper fatigue status did not significantly influence the performance response to this tapering strategy.

## Figures and Tables

**Table 1:** Individual variations (**∆**) of physiological, psychological and biomechanical parameters between T0 and T1.

	Physiological Profile			Psychological Profile				Biomechanical Profile		Decision Making	
Participant	∆ Resting Heart Rate	∆ Exercise Heart Rate	∆ Delta 60	∆ Fatigue	∆ Vigor	∆ EI	EI T1 < 0	∆ Force	∆ Impulsion	Scenario	Group
1			↑↑					↓↓↓↓	↓	1	AF
2					↓↓↓		✔	-	↓↓↓↓	2	F-OR
3		↓↓						↓↓↓	↓↓↓	1	AF
4								↓↓↓↓	↓↓↓	1	AF
5	-	↓↓↓	-	↑↑			✔	↓↓↓	↓↓↓	2	F-OR
6			↑							1	AF
7	↑↑	↓↓		↑↑			✔		-	1	AF
8		↓						↓↓↓	↓↓	1	AF
9							✔	↓↓↓	-	2	F-OR
10			↑↑↑↑							1	AF
11		↓↓↓↓		↑↑↑↑	↓↓↓↓	↓↓↓↓	✔	-	-	2	F-OR
12	↑									1	AF
13			↑↑↑↑	↑↑↑	↓↓↓↓	↓↓↓↓	✔			2	F-OR
14	-	↓↓↓		↑↑↑	↓	↓↓↓	✔		-	2	F-OR
15			↑		↓↓↓↓	-	✔	↓	-	1	AF
16	↑↑↑↑	↓↓	↑	↑↑↑↑					↓↓	2	F-OR
17		↓↓	↑↑↑	↑↑↑		↓↓				2	F-OR
18	↑↑↑	↓↓↓↓		↑↑	↓↓↓↓	↓↓↓↓	✔	-	↓	2	F-OR
19		↓↓↓↓	-							1	AF
20		↓↓↓			↓↓↓↓	↓↓	✔	↓↓↓↓	↓↓↓↓	2	F-OR
21	↑↑↑↑	↓	↑↑↑		↓↓					1	AF
22	-	↓↓↓↓			↓↓↓↓	↓↓↓	✔	↓↓	↓↓	2	F-OR
23	↑		↑↑↑				✔			2	F-OR
24	↑↑	↓↓↓	↑↑		↓↓					1	AF
25		↓↓↓↓	↑↑	↑↑↑	↓↓	↓↓↓	✔			2	F-OR
26	↑↑↑	↓						-		1	AF
27	↑↑↑↑	↓↓		↑↑↑		↓↓↓	✔		↓↓	2	F-OR

AF, acute fatigue; F-OR, functional overreaching; EI, energy index; -, trivial negative change; one arrow, small negative change; two arrows, moderate negative change; three arrows, large negative change; and four arrows, very large negative change (large and very large negative changes are highlighted by grey-shaded cells). Upward arrow: increase, Downward arrow: decrease, Check mark: the situation is observed.

**Table 2:** Characteristics of the acute fatigue (AF) and functional overreaching (F-OR) groups.

	AF Group	F-OR Group
N	13	14
Female (%)	62	50
Age (years old)	16.6 ± 1.6	16.8 ± 1.4
Body height (m)	1.73 ± 0.09	1.76 ± 0.05
Body mass (kg)	63.2 ± 11.6	65.0 ± 6.4
Personal best performance (% world record)	88.3 ± 2.9	89.1 ± 3.1

**Table 3:** Sleep characteristics at T1 and T2 in acute fatigue (AF) and functional overreaching (F-OR) groups. Data are reported as mean ± SD.

	T1		T2	
	AF Group	F-OR Group	AF Group	F-OR Group
***Sleep diary***				
Bedtime (hh:min)	21:44 ± 00:49	22:17 ± 00:58	21:27 ± 00:35	22:02 ± 00:30
Lights out time (hh:min)	22:26 ± 01:07	22:52 ± 00:56	22:06 ± 00:45	22:32 ± 00:32
Wake-up time (hh:min)	07:17 ± 00:37	07:13 ± 00:31	06:50 ± 00:22	07:01 ± 00:36 *^M^
Time from lights out to wake-up (min)	530 ± 74	501 ± 58	524 ± 45	509 ± 60
Spiegel questionnaire score	21.07 ± 3.58	21.35 ± 1.95	20.80 ± 3.14	22.29 ± 2.23
***Accelerometer***				
Latency (min)	12.45 ± 5.23	9.93 ± 3.29	3.64 ± 3.28	5.04 ± 4.15 *^L^
Sleep onset time (hh:min)	22:39 ± 01:05	23:02 ± 00:57	22:10 ± 00:44	22:37 ± 00:31
Total counts, 3 axes per minute from lights out to wake-up (arbitrary units)	280 ± 78	247 ± 54	273 ± 84	246 ± 61
Total sleep time (min)	436 ± 67	431 ± 61	434 ± 41	433 ± 50
Efficiency (%)	82.17 ± 6.93	86.06 ± 3.85	82.89 ± 5.30	85.42 ± 3.80
Movement index (%)	16.22 ± 3.78	15.03 ± 3.10	15.85 ± 2.99	15.06 ± 3.02
Fragmentation index (%)	12.59 ± 5.39	8.39 ± 3.16	13.61 ± 4.36	11.11 ± 2.57

*, significant period effect with ^M^ 0.5 ≤ g< 0.8 or ^L^ g ≥ 0.8. T1: the week preceding the taper period; T2: the taper period.

## Data Availability

The data underlying this study may be made available upon written request to the corresponding author. Requests must clearly specify the type of data requested, the intended purpose of their use, and provide sufficient justification for the request. Data will be shared only to the extent permitted by applicable legal, ethical, and confidentiality requirements. The corresponding author reserves the right to decline any request that lacks adequate justification or whose intended use is considered inappropriate or inconsistent.

## Abbreviations

The following abbreviations are used in this manuscript:

AF	Acute fatigue
EI	Energy index
F-OR	Functional overreaching
LO	Lights out
PBC	Partial-body cryostimulation
WBC	Whole-body cryostimulation

## Appendix A

1. Laboratoire MOVE (UR 20296), UFR STAPS Poitiers, Université de Poitiers, France: Arc-Chagnaud Coralie, Bosquet Laurent, Bretonneau Quentin, Delpech Nathalie, Dugué Benoit, Dupuy Olivier, Enéa Carina, Pichon Aurélien, Tanneau Maxence et Theurot Dimitri.
2. Institut Pprime, UPR 3346, CNRS—Université de Poitiers—ISAE-ENSMA, France: Couvertier Marien, Decatoire Arnaud, Monnet Tony et Samson Mathias.
3. Institut de recherche biomédicale des armées (IRBA), Brétigny-sur-Orge, France: Sauvet Fabien.
4. Unité Vigilance, Fatigue, Sommeil et Santé Publique (VIFASOM, EA7330), Université de Paris, Hôtel Dieu, Paris, France: Sauvet Fabien.
5. Laboratoire SEP (EA 7370), INSEP, France: Morales-Artacho Antonio, Nédelec Mathieu, Pasquier Florane, Poignard Mathilde et Ruffault Alexis.
6. Fédération Française de Natation, Clichy, France: Pla Robin.
7. Laboratoire Culture, Sport, Santé, Société (C3S, EA4660), UFR STAPS Besançon, Université de Franche-Comté, France: Bouzigon Romain.
8. Société Aurore Concept, Noisiel, France: Bouzigon Romain.
9. Société Inside the Athletes 3.0, Besançon, France: Bouzigon Romain.
